# Supplementary material for: RAPID-DASH: Fast and Efficient Assembly of Guide RNA Arrays for Multiplexed CRISPR-Cas9 Applications
Source: bioRxiv. 2025 Sep 18:2025.04.09.648054. Originally published 2025 Apr 9. Preprint. [Version 2] doi: 10.1101/2025.04.09.648054 (PMC12027327; doi:10.1101/2025.04.09.648054)
Supplement: Supplement 1 [file media-1.pdf]

Page 1 Consensus Sequence from Nanopore Sequencing

```
               1                                                                                82
Template       ttgagatcctttttttctgcgcgtaatctgctgcttgcaaacaaaaaaaccaccgctaccagcggtggtttgtttgccggat
Clonal Se...   TTGAGATCCTTTTTTTCTGCGCGTAATCTGCTGCTTGCAAACAAAAAAACCACCGCTACCAGCGGTGGTTTGTTTGCCGGAT

..................................................................................................

               83                                                                              164
Template       caagagctaccaactctttttccgaaggtaactggcttcagcagagcgcagataccaaatactgttcttctagtgtagccgt
Clonal Se...   CAAGAGCTACCAACTCTTTTTCCGAAGGTAACTGGCTTCAGCAGAGCGCAGATACCAAATACTGTTCTTCTAGTGTAGCCGT

..................................................................................................

               165                                                                             246
Template       agttaggccaccacttcaagaactctgtagcaccgcctacatacctcgctctgctaatcctgttaccagtggctgctgccag
Clonal Se...   AGTTAGGCCACCACTTCAAGAACTCTGTAGCACCGCCTACATACCTCGCTCTGCTAATCCTGTTACCAGTGGCTGCTGCCAG

..................................................................................................

               247                                                                             328
Template       tggcgataagtcgtgtcttaccgggttggactcaagacgatagttaccggataaggcgcagcggtcgggctgaacggggggt
Clonal Se...   TGGCGATAAGTCGTGTCTTACCGGGTTGGACTCAAGACGATAGTTACCGGATAAGGCGCAGCGGTCGGGCTGAACGGGGGGT

..................................................................................................

               329                                                                             410
Template       tcgtgcacacagcccagcttggagcgaacgacctacaccgaactgagatacctacagcgtgagctatgagaaagcgccacgc
Clonal Se...   TCGTGCACACAGCCCAGCTTGGAGCGAACGACCTACACCGAACTGAGATACCTACAGCGTGAGCTATGAGAAAGCGCCACGC

..................................................................................................

               411                                                                             492
Template       ttcccgaagggagaaaggcggacaggtatccggtaagcggcagggtcggaacaggagagcgcacgagggagcttccaggggg
Clonal Se...   TTCCCGAAGGGAGAAAGGCGGACAGGTATCCGGTAAGCGGCAGGGTCGGAACAGGAGAGCGCACGAGGGAGCTTCCAGGGGG

..................................................................................................

               493                                                                             574
Template       aaacgcctggtatctttatagtcctgtcgggtttcgccacctctgacttgagcgtcgatttttgtgatgctcgtcagggggg
Clonal Se...   AAACGCCTGGTATCTTTATAGTCCTGTCGGGTTTCGCCACCTCTGACTTGAGCGTCGATTTTTGTGATGCTCGTCAGGGGGG

..................................................................................................

               575                                                                             656
Template       cggagcctatggaaaaacgccagcaacgcggcctttttacggttcctggccttttgctggccttttgctcacatgttctttc
Clonal Se...   CGGAGCCTATGGAAAAACGCCAGCAACGCGGCCTTTTTACGGTTCCTGGCCTTTTGCTGGCCTTTTGCTCACATGTTCTTTC

..................................................................................................

               657                                                                             738
Template       ctgcgttatcccctgattctgtggataaccgtattaccgcctttgagtgagctgataccgctcgccgcagccgaacgaccga
Clonal Se...   CTGCGTTATCCCCTGATTCTGTGGATAACCGTATTACCGCCTTTGAGTGAGCTGATACCGCTCGCCGCAGCCGAACGACCGA

..................................................................................................

               739                                                                             820
Template       gcgcagcgagtcagtgagcgaggaagcggaagagcgcccaatacgcaaaccgcctctccccgcgcgttggccgattcattaa
Clonal Se...   GCGCAGCGAGTCAGTGAGCGAGGAAGCGGAAGAGCGCCCAATACGCAAACCGCCTCTCCCCGCGCGTTGGCCGATTCATTAA

..................................................................................................

               821                                                                             902
Template       tgcagctggcacgacaggtttcccgactggaaagcgggcagtgagcgcaacgcaattaatacgcgtaccgctagccaggaag
Clonal Se...   TGCAGCTGGCACGACAGGTTTCCCGACTGGAAAGCGGGCAGTGAGCGCAACGCAATTAATACGCGTACCGCTAGCCAGGAAG

..................................................................................................
```

```
                903                                                                            984
Template        agtttgtagaaacgcaaaaaggccatccgtcaggatggccttctgcttagtttgatgcctggcagtttatggcgggcgtcct
Clonal Se...    AGTTTGTAGAAACGCAAAAAGGCCATCCGTCAGGATGGCCTTCTGCTTAGTTTGATGCCTGGCAGTTTATGGCGGGCGTCCT

..................................................................................................

                985                                                                           1066
Template        gcccgccaccctccgggccgttgcttcacaacgttcaaatccgctcccggcggatttgtcctactcaggagagcgttcaccg
Clonal Se...    GCCCGCCACCCTCCGGGCCGTTGCTTCACAACGTTCAAATCCGCTCCCGGCGGATTTGTCCTACTCAGGAGAGCGTTCACCG

..................................................................................................

                1067                                                                          1148
Template        acaaacaacagataaaacgaaaggcccagtcttccgactgagcctttcgttttatttgatgcctggcagttccctactctcg
Clonal Se...    ACAAACAACAGATAAAACGAAAGGCCCAGTCTTCCGACTGAGCCTTTCGTTTTATTTGATGCCTGGCAGTTCCCTACTCTCG

..................................................................................................

                1149                                                                          1230
Template        cgttaacgctagcatggatgttttcccagtcacgacgttgtaaaacgacggccagtcttaagcgtctcatggcctgaccccg
Clonal Se...    CGTTAACGCTAGCATGGATGTTTTCCCAGTCACGACGTTGTAAAACGACGGCCAGTCTTAAGCGTCTCATGGCCTGACCCCG

..................................................................................................

                1231                                                                          1312
Template        gaccaagtggtggggtagtaaaacgacggccagtgagggcctatttcccatgattccttcatatttgcatatacgatacaag
Clonal Se...    GACCAAGTGGTGGGGTAGTAAAACGACGGCCAGTGAGGGCCTATTTCCCATGATTCCTTCATATTTGCATATACGATACAAG

..................................................................................................

                1313                                                                          1394
Template        gctgttagagagataattagaattaatttgactgtaaacacaaagatattagtacaaaatacgtgacgtagaaagtaataat
Clonal Se...    GCTGTTAGAGAGATAATTAGAATTAATTTGACTGTAAACACAAAGATATTAGTACAAAATACGTGACGTAGAAAGTAATAAT

..................................................................................................

                1395                                                                          1476
Template        ttcttgggtagtttgcagttttaaaattatgttttaaaatggactatcatatgcttaccgtaacttgaaagtatttcgattt
Clonal Se...    TTCTTGGGTAGTTTGCAGTTTTAAAATTATGTTTTAAAATGGACTATCATATGCTTACCGTAACTTGAAAGTATTTCGATTT

..................................................................................................

                1477                                                                          1558
Template        cttggctttatatatcttgtggaaaggacgaaacaccgggaatcccttctgcagcaccgttttagagctagaaatagcaagt
Clonal Se...    CTTGGCTTTATATATCTTGTGGAAAGGACGAAACACCGGGAATCCCTTCTGCAGCACCGTTTTAGAGCTAGAAATAGCAAGT

..................................................................................................

                1559                                                                          1640
Template        taaaataaggctagtccgttatcaacttgaaaaagtggcaccgagtcggtgctttttttcatggtcatagctgtttccttag
Clonal Se...    TAAAATAAGGCTAGTCCGTTATCAACTTGAAAAAGTGGCACCGAGTCGGTGCTTTTTTTCATGGTCATAGCTGTTTCCTTAG

..................................................................................................

                1641                                                                          1722
Template        agtaaaacgacggccagtgagggcctatttcccatgattccttcatatttgcatatacgatacaaggctgttagagagataa
Clonal Se...    AGTAAAACGACGGCCAGTGAGGGCCTATTTCCCATGATTCCTTCATATTTGCATATACGATACAAGGCTGTTAGAGAGATAA

..................................................................................................

                1723                                                                          1804
Template        ttagaattaatttgactgtaaacacaaagatattagtacaaaatacgtgacgtagaaagtaataatttcttgggtagtttgc
Clonal Se...    TTAGAATTAATTTGACTGTAAACACAAAGATATTAGTACAAAATACGTGACGTAGAAAGTAATAATTTCTTGGGTAGTTTGC

..................................................................................................
```

```
               1805                                                                          1886
Template       agttttaaaattatgttttaaaatggactatcatatgcttaccgtaacttgaaagtatttcgatttcttggctttatatatc
Clonal Se...   AGTTTTAAAATTATGTTTTAAAATGGACTATCATATGCTTACCGTAACTTGAAAGTATTTCGATTTCTTGGCTTTATATATC

.................................................................................................

               1887                                                                          1968
Template       ttgtggaaaggacgaaacaccggagtccgagcagaagaagaagttttagagctagaaatagcaagttaaaataaggctagtc
Clonal Se...   TTGTGGAAAGGACGAAACACCGGAGTCCGAGCAGAAGAAGAAGTTTTAGAGCTAGAAATAGCAAGTTAAAATAAGGCTAGTC

.................................................................................................

               1969                                                                          2050
Template       cgttatcaacttgaaaaagtggcaccgagtcggtgctttttttcatggtcatagctgtttcctctccgtaaaacgacggcca
Clonal Se...   CGTTATCAACTTGAAAAAGTGGCACCGAGTCGGTGCTTTTTTTCATGGTCATAGCTGTTTCCTCTCCGTAAAACGACGGCCA

.................................................................................................

               2051                                                                          2132
Template       gtgagggcctatttcccatgattccttcatatttgcatatacgatacaaggctgttagagagataattagaattaatttgac
Clonal Se...   GTGAGGGCCTATTTCCCATGATTCCTTCATATTTGCATATACGATACAAGGCTGTTAGAGAGATAATTAGAATTAATTTGAC

.................................................................................................

               2133                                                                          2214
Template       tgtaaacacaaagatattagtacaaaatacgtgacgtagaaagtaataatttcttgggtagtttgcagttttaaaattatgt
Clonal Se...   TGTAAACACAAAGATATTAGTACAAAATACGTGACGTAGAAAGTAATAATTTCTTGGGTAGTTTGCAGTTTTAAAATTATGT

.................................................................................................

               2215                                                                          2296
Template       tttaaaatggactatcatatgcttaccgtaacttgaaagtatttcgatttcttggctttatatatcttgtggaaaggacgaa
Clonal Se...   TTTAAAATGGACTATCATATGCTTACCGTAACTTGAAAGTATTTCGATTTCTTGGCTTTATATATCTTGTGGAAAGGACGAA

.................................................................................................

               2297                                                                          2378
Template       acaccgtttatcacaggctccaggaagttttagagctagaaatagcaagttaaaataaggctagtccgttatcaacttgaaa
Clonal Se...   ACACCGTTTATCACAGGCTCCAGGAAGTTTTAGAGCTAGAAATAGCAAGTTAAAATAAGGCTAGTCCGTTATCAACTTGAAA

.................................................................................................

               2379                                                                          2460
Template       aagtggcaccgagtcggtgctttttttcatggtcatagctgtttcctatcagtaaaacgacggccagtgagggcctatttcc
Clonal Se...   AAGTGGCACCGAGTCGGTGCTTTTTTTCATGGTCATAGCTGTTTCCTATCAGTAAAACGACGGCCAGTGAGGGCCTATTTCC

.................................................................................................

               2461                                                                          2542
Template       catgattccttcatatttgcatatacgatacaaggctgttagagagataattagaattaatttgactgtaaacacaaagata
Clonal Se...   CATGATTCCTTCATATTTGCATATACGATACAAGGCTGTTAGAGAGATAATTAGAATTAATTTGACTGTAAACACAAAGATA

.................................................................................................

               2543                                                                          2624
Template       ttagtacaaaatacgtgacgtagaaagtaataatttcttgggtagtttgcagttttaaaattatgttttaaaatggactatc
Clonal Se...   TTAGTACAAAATACGTGACGTAGAAAGTAATAATTTCTTGGGTAGTTTGCAGTTTTAAAATTATGTTTTAAAATGGACTATC

.................................................................................................

               2625                                                                          2706
Template       atatgcttaccgtaacttgaaagtatttcgatttcttggctttatatatcttgtggaaaggacgaaacaccgggcccagact
Clonal Se...   ATATGCTTACCGTAACTTGAAAGTATTTCGATTTCTTGGCTTTATATATCTTGTGGAAAGGACGAAACACCGGGCCCAGACT

.................................................................................................
```

```
             2707                                                                          2788
Template     gagcacgtgagttttagagctagaaatagcaagttaaaataaggctagtccgttatcaacttgaaaaagtggcaccgagtcg
Clonal Se... GAGCACGTGAGTTTTAGAGCTAGAAATAGCAAGTTAAAATAAGGCTAGTCCGTTATCAACTTGAAAAAGTGGCACCGAGTCG

...............................................................................................

             2789                                                                          2870
Template     gtgctttttttcatggtcatagctgtttcctctgagtaaaacgacggccagtgagggcctatttcccatgattccttcatat
Clonal Se... GTGCTTTTTTTCATGGTCATAGCTGTTTCCTCTGAGTAAAACGACGGCCAGTGAGGGCCTATTTCCCATGATTCCTTCATAT

...............................................................................................

             2871                                                                          2952
Template     ttgcatatacgatacaaggctgttagagagataattagaattaatttgactgtaaacacaaagatattagtacaaaatacgt
Clonal Se... TTGCATATACGATACAAGGCTGTTAGAGAGATAATTAGAATTAATTTGACTGTAAACACAAAGATATTAGTACAAAATACGT

...............................................................................................

             2953                                                                          3034
Template     gacgtagaaagtaataatttcttgggtagtttgcagttttaaaattatgttttaaaatggactatcatatgcttaccgtaac
Clonal Se... GACGTAGAAAGTAATAATTTCTTGGGTAGTTTGCAGTTTTAAAATTATGTTTTAAAATGGACTATCATATGCTTACCGTAAC

...............................................................................................

             3035                                                                          3116
Template     ttgaaagtatttcgatttcttggctttatatatcttgtggaaaggacgaaacaccgactcacgctggatagcctccgtttta
Clonal Se... TTGAAAGTATTTCGATTTCTTGGCTTTATATATCTTGTGGAAAGGACGAAACACCGACTCACGCTGGATAGCCTCCGTTTTA

...............................................................................................

             3117                                                                          3198
Template     gagctagaaatagcaagttaaaataaggctagtccgttatcaacttgaaaaagtggcaccgagtcggtgctttttttcatgg
Clonal Se... GAGCTAGAAATAGCAAGTTAAAATAAGGCTAGTCCGTTATCAACTTGAAAAAGTGGCACCGAGTCGGTGCTTTTTTTCATGG

...............................................................................................

             3199                                                                          3280
Template     tcatagctgtttcctagcggtaaaacgacggccagtgagggcctatttcccatgattccttcatatttgcatatacgataca
Clonal Se... TCATAGCTGTTTCCTAGCGGTAAAACGACGGCCAGTGAGGGCCTATTTCCCATGATTCCTTCATATTTGCATATACGATACA

...............................................................................................

             3281                                                                          3362
Template     aggctgttagagagataattagaattaatttgactgtaaacacaaagatattagtacaaaatacgtgacgtagaaagtaata
Clonal Se... AGGCTGTTAGAGAGATAATTAGAATTAATTTGACTGTAAACACAAAGATATTAGTACAAAATACGTGACGTAGAAAGTAATA

...............................................................................................

             3363                                                                          3444
Template     atttcttgggtagtttgcagttttaaaattatgttttaaaatggactatcatatgcttaccgtaacttgaaagtatttcgat
Clonal Se... ATTTCTTGGGTAGTTTGCAGTTTTAAAATTATGTTTTAAAATGGACTATCATATGCTTACCGTAACTTGAAAGTATTTCGAT

...............................................................................................

             3445                                                                          3526
Template     ttcttggctttatatatcttgtggaaaggacgaaacaccggtcatcttagtcattacctggttttagagctagaaatagcaa
Clonal Se... TTCTTGGCTTTATATATCTTGTGGAAAGGACGAAACACCGGTCATCTTAGTCATTACCTGGTTTTAGAGCTAGAAATAGCAA

...............................................................................................

             3527                                                                          3608
Template     gttaaaataaggctagtccgttatcaacttgaaaaagtggcaccgagtcggtgctttttttcatggtcatagctgtttccta
Clonal Se... GTTAAAATAAGGCTAGTCCGTTATCAACTTGAAAAAGTGGCACCGAGTCGGTGCTTTTTTTCATGGTCATAGCTGTTTCCTA

...............................................................................................
```

```
               3609                                                                         3690
Template       agggtaaaacgacggccagtgagggcctatttcccatgattccttcatatttgcatatacgatacaaggctgttagagagat
Clonal Se...   AGGGTAAAACGACGGCCAGTGAGGGCCTATTTCCCATGATTCCTTCATATTTGCATATACGATACAAGGCTGTTAGAGAGAT

.................................................................................................

               3691                                                                         3772
Template       aattagaattaatttgactgtaaacacaaagatattagtacaaaatacgtgacgtagaaagtaataatttcttgggtagttt
Clonal Se...   AATTAGAATTAATTTGACTGTAAACACAAAGATATTAGTACAAAATACGTGACGTAGAAAGTAATAATTTCTTGGGTAGTTT

.................................................................................................

               3773                                                                         3854
Template       gcagttttaaaattatgttttaaaatggactatcatatgcttaccgtaacttgaaagtatttcgatttcttggctttatata
Clonal Se...   GCAGTTTTAAAATTATGTTTTAAAATGGACTATCATATGCTTACCGTAACTTGAAAGTATTTCGATTTCTTGGCTTTATATA

.................................................................................................

               3855                                                                         3936
Template       tcttgtggaaaggacgaaacaccgggcactgcggctggaggtgggttttagagctagaaatagcaagttaaaataaggctag
Clonal Se...   TCTTGTGGAAAGGACGAAACACCGGGCACTGCGGCTGGAGGTGGGTTTTAGAGCTAGAAATAGCAAGTTAAAATAAGGCTAG

.................................................................................................

               3937                                                                         4018
Template       tccgttatcaacttgaaaaagtggcaccgagtcggtgctttttttcatggtcatagctgtttcctcatcgtaaaacgacggc
Clonal Se...   TCCGTTATCAACTTGAAAAAGTGGCACCGAGTCGGTGCTTTTTTTCATGGTCATAGCTGTTTCCTCATCGTAAAACGACGGC

.................................................................................................

               4019                                                                         4100
Template       cagtgagggcctatttcccatgattccttcatatttgcatatacgatacaaggctgttagagagataattagaattaatttg
Clonal Se...   CAGTGAGGGCCTATTTCCCATGATTCCTTCATATTTGCATATACGATACAAGGCTGTTAGAGAGATAATTAGAATTAATTTG

.................................................................................................

               4101                                                                         4182
Template       actgtaaacacaaagatattagtacaaaatacgtgacgtagaaagtaataatttcttgggtagtttgcagttttaaaattat
Clonal Se...   ACTGTAAACACAAAGATATTAGTACAAAATACGTGACGTAGAAAGTAATAATTTCTTGGGTAGTTTGCAGTTTTAAAATTAT

.................................................................................................

               4183                                                                         4264
Template       gttttaaaatggactatcatatgcttaccgtaacttgaaagtatttcgatttcttggctttatatatcttgtggaaaggacg
Clonal Se...   GTTTTAAAATGGACTATCATATGCTTACCGTAACTTGAAAGTATTTCGATTTCTTGGCTTTATATATCTTGTGGAAAGGACG

.................................................................................................

               4265                                                                         4346
Template       aaacaccgcacctacctaagaaccatccgttttagagctagaaatagcaagttaaaataaggctagtccgttatcaacttga
Clonal Se...   AAACACCGCACCTACCTAAGAACCATCCGTTTTAGAGCTAGAAATAGCAAGTTAAAATAAGGCTAGTCCGTTATCAACTTGA

.................................................................................................

               4347                                                                         4428
Template       aaaagtggcaccgagtcggtgctttttttcatggtcatagctgtttcctacctgtaaaacgacggccagtgagggcctattt
Clonal Se...   AAAAGTGGCACCGAGTCGGTGCTTTTTTTCATGGTCATAGCTGTTTCCTACCTGTAAAACGACGGCCAGTGAGGGCCTATTT

.................................................................................................

               4429                                                                         4510
Template       cccatgattccttcatatttgcatatacgatacaaggctgttagagagataattagaattaatttgactgtaaacacaaaga
Clonal Se...   CCCATGATTCCTTCATATTTGCATATACGATACAAGGCTGTTAGAGAGATAATTAGAATTAATTTGACTGTAAACACAAAGA

.................................................................................................
```

```
             4511                                                                             4592
Template     tattagtacaaaatacgtgacgtagaaagtaataatttcttgggtagtttgcagttttaaaattatgttttaaaatggacta
Clonal Se... TATTAGTACAAAATACGTGACGTAGAAAGTAATAATTTCTTGGGTAGTTTGCAGTTTTAAAATTATGTTTTAAAATGGACTA

..................................................................................................

             4593                                                                             4674
Template     tcatatgcttaccgtaacttgaaagtatttcgatttcttggctttatatatcttgtggaaaggacgaaacaccgttcgtatc
Clonal Se... TCATATGCTTACCGTAACTTGAAAGTATTTCGATTTCTTGGCTTTATATATCTTGTGGAAAGGACGAAACACCGTTCGTATC

..................................................................................................

             4675                                                                             4756
Template     tgtaaaaccaaggttttagagctagaaatagcaagttaaaataaggctagtccgttatcaacttgaaaaagtggcaccgagt
Clonal Se... TGTAAAACCAAGGTTTTAGAGCTAGAAATAGCAAGTTAAAATAAGGCTAGTCCGTTATCAACTTGAAAAAGTGGCACCGAGT

..................................................................................................

             4757                                                                             4838
Template     cggtgctttttttcatggtcatagctgtttcctgcgagtaaaacgacggccagtgagggcctatttcccatgattccttcat
Clonal Se... CGGTGCTTTTTTTCATGGTCATAGCTGTTTCCTGCGAGTAAAACGACGGCCAGTGAGGGCCTATTTCCCATGATTCCTTCAT

..................................................................................................

             4839                                                                             4920
Template     atttgcatatacgatacaaggctgttagagagataattagaattaatttgactgtaaacacaaagatattagtacaaaatac
Clonal Se... ATTTGCATATACGATACAAGGCTGTTAGAGAGATAATTAGAATTAATTTGACTGTAAACACAAAGATATTAGTACAAAATAC

..................................................................................................

             4921                                                                             5002
Template     gtgacgtagaaagtaataatttcttgggtagtttgcagttttaaaattatgttttaaaatggactatcatatgcttaccgta
Clonal Se... GTGACGTAGAAAGTAATAATTTCTTGGGTAGTTTGCAGTTTTAAAATTATGTTTTAAAATGGACTATCATATGCTTACCGTA

..................................................................................................

             5003                                                                             5084
Template     acttgaaagtatttcgatttcttggctttatatatcttgtggaaaggacgaaacaccgcacggtcaccctgacacgctgttt
Clonal Se... ACTTGAAAGTATTTCGATTTCTTGGCTTTATATATCTTGTGGAAAGGACGAAACACCGCACGGTCACCCTGACACGCTGTTT

..................................................................................................

             5085                                                                             5166
Template     tagagctagaaatagcaagttaaaataaggctagtccgttatcaacttgaaaaagtggcaccgagtcggtgctttttttcat
Clonal Se... TAGAGCTAGAAATAGCAAGTTAAAATAAGGCTAGTCCGTTATCAACTTGAAAAAGTGGCACCGAGTCGGTGCTTTTTTTCAT

..................................................................................................

             5167                                                                             5248
Template     ggtcatagctgtttcctcgtagcaagcaagcgctcgaaacggtgcagcggctgttgccggtgctgtgccaggaccatggcct
Clonal Se... GGTCATAGCTGTTTCCTCGTAGCAAGCAAGCGCTCGAAACGGTGCAGCGGCTGTTGCCGGTGCTGTGCCAGGACCATGGCCT

..................................................................................................

             5249                                                                             5330
Template     gaccccggaccaagtggtggctatcgagacgtctagaccagccaggacagaaatgcctcgacttcgctgctacccaaggttg
Clonal Se... GACCCCGGACCAAGTGGTGGCTATCGAGACGTCTAGACCAGCCAGGACAGAAATGCCTCGACTTCGCTGCTACCCAAGGTTG

..................................................................................................

             5331                                                                             5412
Template     ccgggtgacgcacaccgtggaaacggatgaaggcacgaacccagtggacataagcctgttcggttcgtaagctgtaatgcaa
Clonal Se... CCGGGTGACGCACACCGTGGAAACGGATGAAGGCACGAACCCAGTGGACATAAGCCTGTTCGGTTCGTAAGCTGTAATGCAA

..................................................................................................
```

```
             5413                                                                          5494
Template     gtagcgtatgcgctcacgcaactggtccagaaccttgaccgaacgcagcggtggtaacggcgcagtggcggttttcatggct
Clonal Se... GTAGCGTATGCGCTCACGCAACTGGTCCAGAACCTTGACCGAACGCAGCGGTGGTAACGGCGCAGTGGCGGTTTTCATGGCT

...............................................................................................

             5495                                                                          5576
Template     tgttatgactgttttttggggtacagtctatgcctcgggcatccaagcagcaagcgcgttacgccgtgggtcgatgtttga
Clonal Se... TGTTATGACTGTTTTTTGGGGTACAGTCTATGCCTCGGGCATCCAAGCAGCAAGCGCGTTACGCCGTGGGTCGATGTTTGA

...............................................................................................

             5577                                                                          5658
Template     tgttatggagcagcaacgatgttacgcagcagggcagtcgccctaaaacaaagttaaacattatgagggaagcggtgatcgc
Clonal Se... TGTTATGGAGCAGCAACGATGTTACGCAGCAGGGCAGTCGCCCTAAAACAAAGTTAAACATTATGAGGGAAGCGGTGATCGC

...............................................................................................

             5659                                                                          5740
Template     cgaagtatcgactcaactatcagaggtagttggcgtcatcgagcgccatctcgaaccgacgttgctggccgtacatttgtac
Clonal Se... CGAAGTATCGACTCAACTATCAGAGGTAGTTGGCGTCATCGAGCGCCATCTCGAACCGACGTTGCTGGCCGTACATTTGTAC

...............................................................................................

             5741                                                                          5822
Template     ggctccgcagtggatggcggcctgaagccacacagtgatattgatttgctggttacggtgaccgtaaggcttgatgaaacaa
Clonal Se... GGCTCCGCAGTGGATGGCGGCCTGAAGCCACACAGTGATATTGATTTGCTGGTTACGGTGACCGTAAGGCTTGATGAAACAA

...............................................................................................

             5823                                                                          5904
Template     cgcggcgagctttgatcaacgaccttttggaaacttcggcttcccctggagagagcgagattctccgcgctgtagaagtcac
Clonal Se... CGCGGCGAGCTTTGATCAACGACCTTTTGGAAACTTCGGCTTCCCCTGGAGAGAGCGAGATTCTCCGCGCTGTAGAAGTCAC

...............................................................................................

             5905                                                                          5986
Template     cattgttgtgcacgacgacatcattccgtggcgttatccagctaagcgcgaactgcaatttggagaatggcagcgcaatgac
Clonal Se... CATTGTTGTGCACGACGACATCATTCCGTGGCGTTATCCAGCTAAGCGCGAACTGCAATTTGGAGAATGGCAGCGCAATGAC

...............................................................................................

             5987                                                                          6068
Template     attcttgcaggtatcttcgagccagccacgatcgacattgatctggctatcttgctgacaaaagcaagagaacatagcgttg
Clonal Se... ATTCTTGCAGGTATCTTCGAGCCAGCCACGATCGACATTGATCTGGCTATCTTGCTGACAAAAGCAAGAGAACATAGCGTTG

...............................................................................................

             6069                                                                          6150
Template     ccttggtaggtccagcggcggaggaactctttgatccggttcctgaacaggatctatttgaggcgctaaatgaaaccttaac
Clonal Se... CCTTGGTAGGTCCAGCGGCGGAGGAACTCTTTGATCCGGTTCCTGAACAGGATCTATTTGAGGCGCTAAATGAAACCTTAAC

...............................................................................................

             6151                                                                          6232
Template     gctatggaactcgccgcccgactgggctggcgatgagcgaaatgtagtgcttacgttgtcccgcatttggtacagcgcagta
Clonal Se... GCTATGGAACTCGCCGCCCGACTGGGCTGGCGATGAGCGAAATGTAGTGCTTACGTTGTCCCGCATTTGGTACAGCGCAGTA

...............................................................................................

             6233                                                                          6314
Template     accggcaaaatcgcgccgaaggatgtcgctgccgactgggcaatggagcgcctgccggcccagtatcagcccgtcatacttg
Clonal Se... ACCGGCAAAATCGCGCCGAAGGATGTCGCTGCCGACTGGGCAATGGAGCGCCTGCCGGCCCAGTATCAGCCCGTCATACTTG

...............................................................................................
```

```
             6315                                                                         6396
Template     aagctagacaggcttatcttggacaagaagaagatcgcttggcctcgcgcgcagatcagttggaagaatttgtccactacgt
Clonal Se... AAGCTAGACAGGCTTATCTTGGACAAGAAGAAGATCGCTTGGCCTCGCGCGCAGATCAGTTGGAAGAATTTGTCCACTACGT

.........................................................................................

             6397                                                                         6478
Template     gaaaggcgagatcaccaaggtagtcggcaaataaccctcgagccacccatgaccaaaatcccttaacgtgagttacgcgtcg
Clonal Se... GAAAGGCGAGATCACCAAGGTAGTCGGCAAATAACCCTCGAGCCACCCATGACCAAAATCCCTTAACGTGAGTTACGCGTCG

.........................................................................................

             6479                                        6522
Template     ttccactgagcgtcagaccccgtagaaaagatcaaaggatcttc
Clonal Se... TTCCACTGAGCGTCAGACCCCGTAGAAAAGATCAAAGGATCTTC

.........................................................................................
```
