# Supplementary material for: RAPID-DASH: Single-Day Assembly of Guide RNA Arrays for Multiplexed CRISPR-Cas9 Applications
Source: bioRxiv. 2025 Apr 9:2025.04.09.648054. Preprint. [Version 1] doi: 10.1101/2025.04.09.648054 (PMC12027327; doi:10.1101/2025.04.09.648054)
Supplement: Supplementary file 1 [file media-1.pdf]

RAPID-DASH: Single-Day Assembly of Guide RNA Arrays for Multiplexed CRISPR-Cas9 Applications

Supplementary Materials

This document includes the following:

Supplementary figure 1

Supplementary table 1

Oligos and sequences for gRNA assembly

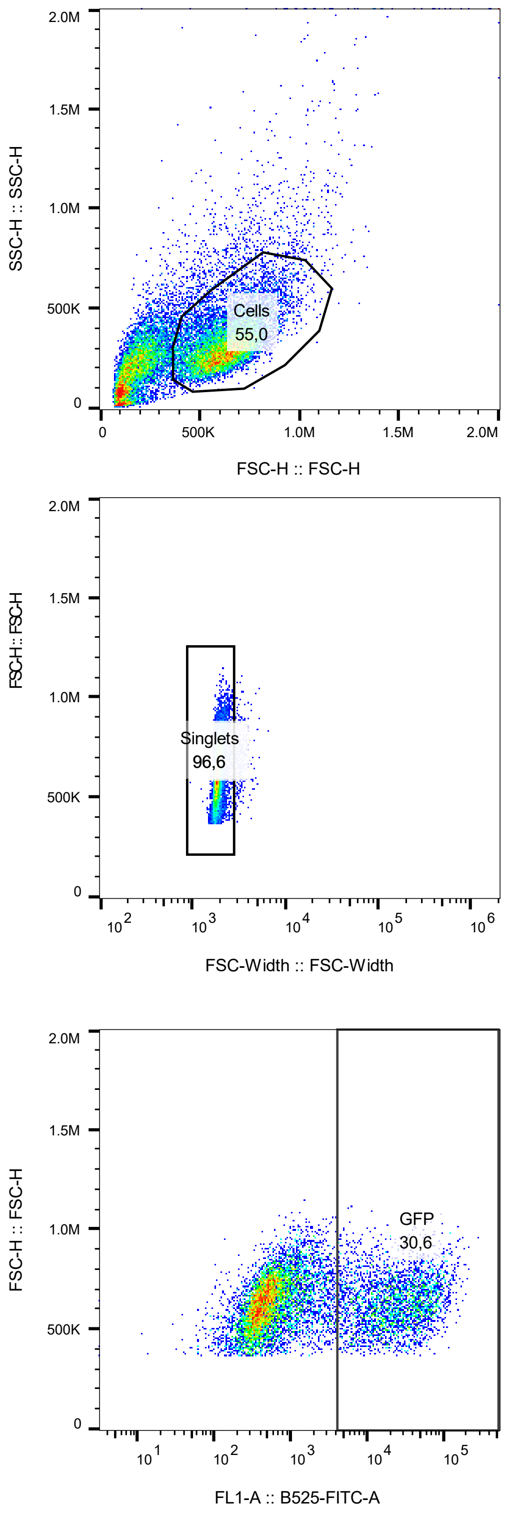

Supplementary Figure 1: Gating summary for flow cytometry data. Cells were gated for singlets and then GFP positive cells using FlowJo (v10).

Supplementary Table 1: gRNA array assembly efficiency

Number of gRNAs in the array	Assembly 1	Assembly 2	Assembly 3
**0**	0	0	0
**1**	2	0	0
**2**	0	0	7
**3**	0	0	5
**4**	1	1	8
**5**	0	1	11
**6**	2	1	1
**7**	0	3	0
**8**	3	3	3
**9**	1	2	6
**10**	54	150	141
**>10**	0	1	2
**Not in any range**	3	9	6
**Total**	**66**	**171**	**190**
**% Correct**	**81.82**	**87.72**	**74.21**

Supplementary Table 2: Read length binning thresholds for calculating the number of gRNAs in an array

Number of gRNAs	Expected Length (bp)	Lower range (bp)	Upper Range (bp)
>10	>4311	4241	
10	3917	3847	3987
9	3523	3453	3593
8	3129	3059	3199
7	2735	2665	2805
6	2341	2271	2411
5	1947	1877	2017
4	1553	1483	1623
3	1159	1089	1229
2	765	695	835
1	371	301	441
0	0	0	70

Oligos and sequences for gRNA assembly

U6 Promoter

Forward primer: GTAAAACGACGGCCAGTgagggcctatttcccatgattc

Reverse primer: GGTGTTTCGTCCTTTCCAC

Amplicon sequence:
GTAAAACGACGGCCAGTgagggcctatttcccatgattccttcatatttgcatatacgatacaaggctgttagagagataattggaattaatttgactgtaaacacaaagatattagtacaaaatacgtgacgtagaaagtaataatttcttgggtagtttgcagttttaaaattatgttttaaaatggactatcatatgcttaccgtaacttgaaagtatttcgatttcttggctttatatatcttGTGGAAAGGACGAAACACC

gRNA terminator scaffold

Forward primer: gttttagagctaGAAAtagcaag

Reverse primer: AGGAAACAGCTATGACCATGAAAAAAAgcaccgactcggtgccac

Amplicon sequence:
gttttagagctaGAAAtagcaagttaaaataaggctagtccgttatcaacttgaaaaagtggcaccgagtcggtgcTTTTTTTCATGGTCATAGCTGTTTCCT

gRNA spacer oligos

Sample:
GTGGAAAGGACGAAACACCgNNNNNNNNNNNNNNNNNNNNgttttagagctaGAAAtag

	gRNA spacer	Ordered oligo
1	GGAATCCCTTCTGCAGCACC	GTGGAAAGGACGAAACACCgGGAATCCCTTCTGCAGCACCgttttagagctaGAAAtag
2	GAGTCCGAGCAGAAGAAGAA	GTGGAAAGGACGAAACACCgGAGTCCGAGCAGAAGAAGAAgttttagagctaGAAAtag
3	TTTATCACAGGCTCCAGGAA	GTGGAAAGGACGAAACACCgTTTATCACAGGCTCCAGGAAgttttagagctaGAAAtag
4	GGCCCAGACTGAGCACGTGA	GTGGAAAGGACGAAACACCgGGCCCAGACTGAGCACGTGAgttttagagctaGAAAtag
5	ACTCACGCTGGATAGCCTCC	GTGGAAAGGACGAAACACCgACTCACGCTGGATAGCCTCCgttttagagctaGAAAtag
6	GTCATCTTAGTCATTACCTG	GTGGAAAGGACGAAACACCgGTCATCTTAGTCATTACCTGgttttagagctaGAAAtag
7	GGCACTGCGGCTGGAGGTGG	GTGGAAAGGACGAAACACCgGGCACTGCGGCTGGAGGTGGgttttagagctaGAAAtag

8	CACCTACCTAAGAACCATCC	GTGGAAAGGACGAAACACCgCACCTACCTAAGAACCATCCgttttagagctaGAAAtag
9	TTCGTATCTGTAAAACCAAG	GTGGAAAGGACGAAACACCgTTCGTATCTGTAAAACCAAGgttttagagctaGAAAtag
10	GTATCTAGTGTTGGTGTCCT	GTGGAAAGGACGAAACACCgGTATCTAGTGTTGGTGTCCTgttttagagctaGAAAtag
GFP targeting	CACGGTCACCCTGACACGCT	GTGGAAAGGACGAAACACCgCACGGTCACCCTGACACGCTgttttagagctaGAAAtag

Primers for gRNA unit amplification

gRNA unit	Primer direction	Sequence	BsaI Overhang
**1**	**Fwd**	ATAAGGATCCGGTCTCAGGTAGTAAAACGACGGCCAGT	GGTA
**1**	**Rev**	ATAATGTACAGGTCTCTTCTAAGGAAACAGCTATGACCATG	TCTA
**2**	**Fwd**	ATAAGGATCCGGTCTCATAGAGTAAAACGACGGCCAGT	TAGA
**2**	**Rev**	ATAATGTACAGGTCTCTGGAGAGGAAACAGCTATGACCATG	GGAG
**3**	**Fwd**	ATAAGGATCCGGTCTCACTCCGTAAAACGACGGCCAGT	CTCC
**3**	**Rev**	ATAATGTACAGGTCTCTTGATAGGAAACAGCTATGACCATG	TGAT
**4**	**Fwd**	ATAAGGATCCGGTCTCAATCAGTAAAACGACGGCCAGT	ATCA
**4**	**Rev**	ATAATGTACAGGTCTCTTCAGAGGAAACAGCTATGACCATG	TCAG
**5**	**Fwd**	ATAAGGATCCGGTCTCACTGAGTAAAACGACGGCCAGT	CTGA
**5**	**Rev**	ATAATGTACAGGTCTCTCGCTAGGAAACAGCTATGACCATG	CGCT
**6**	**Fwd**	ATAAGGATCCGGTCTCAAGCGGTAAAACGACGGCCAGT	AGCG
**6**	**Rev**	ATAATGTACAGGTCTCTCCTTAGGAAACAGCTATGACCATG	CCTT
**7**	**Fwd**	ATAAGGATCCGGTCTCAAAGGGTAAAACGACGGCCAGT	AAGG
**7**	**Rev**	ATAATGTACAGGTCTCTGATGAGGAAACAGCTATGACCATG	GATG
**8**	**Fwd**	ATAAGGATCCGGTCTCACATCGTAAAACGACGGCCAGT	CATC
**8**	**Rev**	ATAATGTACAGGTCTCTAGGTAGGAAACAGCTATGACCATG	AGGT
**9**	**Fwd**	ATAAGGATCCGGTCTCAACCTGTAAAACGACGGCCAGT	ACCT
**9**	**Rev**	ATAATGTACAGGTCTCTTCGCAGGAAACAGCTATGACCATG	TCGC
**10**	**Fwd**	ATAAGGATCCGGTCTCAGCGAGTAAAACGACGGCCAGT	GCGA
**10**	**Rev**	ATAATGTACAGGTCTCTTACGAGGAAACAGCTATGACCATG	TACG
